# Outcome Reporting in Studies Investigating Treatment for Caesarean Scar Ectopic Pregnancy: A Systematic Review

**DOI:** 10.1111/1471-0528.17989

**Published:** 2024-11-07

**Authors:** Simrit Nijjar, Simarjit Sandhar, Ilan E. Timor‐Tritsch, Andrea Kaelin Agten, Jin Li, Krystle Y. Chong, Munira Oza, Rosanna Acklom, Francesco D'Antonio, Lan N. Vuong, Ben Mol, Cecilia Bottomley, Davor Jurkovic, the International Collaboration COSCAR

**Affiliations:** ^1^ EGA Institute for Women's Health, Faculty of Population Health Sciences University College London London UK; ^2^ Hackensack Meridian School of Medicine Nutley New Jersey USA; ^3^ Liverpool Women's Hospital NHS Foundation Trust Liverpool UK; ^4^ National Clinical Research Center for Obstetric and Gynecologic Diseases, Department of Obstetrics and Gynecology, Peking Union Medical College Hospital Chinese Academy of Medical Sciences and Peking Union Medical College Beijing China; ^5^ Department of Obstetrics and Gynaecology Monash University Clayton Victoria Australia; ^6^ The Ectopic Pregnancy Trust London UK; ^7^ Center for Fetal Care and High‐Risk Pregnancy, Department of Obstetrics and Gynecology University Hospital of Chieti Chieti Italy; ^8^ Department of Obstetrics and Gynecology University of Medicine and Pharmacy at Ho Chi Minh City Ho Chi Minh City Vietnam; ^9^ Monash Women's, Monash Health Clayton Victoria Australia

**Keywords:** caesarean scar ectopic, core outcome sets, outcome reporting, outcome variation

## Abstract

**Background:**

Caesarean scar ectopic pregnancy (CSEP) is associated with significant maternal and foetal morbidity. However, the optimal treatment remains unknown.

**Objectives:**

The aim of this study was to review outcomes reported in studies on CSEP treatment and outcome reporting quality.

**Search Strategy:**

We reviewed 1270 articles identified through searching PubMed, MEDLINE and Google Scholar from 2014 to 2024 using the search terms ‘caesarean scar ectopic pregnancy and caesarean scar pregnancy’.

**Selection Criteria:**

We included all study types evaluating any form of CSEP treatment, with a sample size of ≥ 50, where diagnosis was described, and the article was in English.

**Data Collection and Analysis:**

Two authors independently reviewed studies and assessed outcome reporting and methodological quality. The relationship between outcome reporting quality and publication year and journal type was assessed with univariate and bivariate models.

**Main Results:**

A total of 108 studies, including 17 941 women, were included. 83% of all studies originated from China. Studies reported on 326 outcomes; blood loss (86%), need for additional intervention (77%) and time for serum hCG to normalise post treatment (69%) were the most common outcomes. A primary outcome was clearly defined in 11 (10%) studies. The median quality of outcome reporting was 3 (IQR 3–4). No relationship was demonstrated between outcome reporting quality and publication year (*p* = 0.116) or journal type (*p* = 0.503).

**Conclusions:**

This review demonstrates that there is a wide variation in outcomes reported in studies on CSEP treatment. Development and implementation of a core outcome set by international stakeholders which includes patients is urgently needed to enable high‐quality research that is both useful and relevant to patients.

## 1 Introduction

Caesarean scar ectopic pregnancy (CSEP), also referred to as Caesarean scar pregnancy, used to be considered rare, but it is now the most commonly reported type of uterine ectopic pregnancy. It has been reported that as many as 1 in 531 women with previous caesarean delivery (CD) go on to develop a CSEP [1]. This increase in the incidence of CSEP is likely due to increased awareness of the condition, as well as increased vigilance in diagnosis, although there is still a long way to go as several different diagnostic criteria and classification systems remain in use, with no evidence of superiority of any given system [2]. CSEP has been associated with significant maternal and foetal morbidity both with continuing the pregnancy but also terminating the pregnancy [3, 4, 5]. Despite this recognised morbidity, there remains no consensus on the optimal treatment approach [3, 6].

Several systematic reviews and more recently a network meta‐analysis have reported between them 14 and 17 different treatment modalities across 52–73 studies, ranging from surgical, medical, minimally invasive approaches (uterine artery embolisation—‘UAE’, high intensity focused ultrasound—‘HIFU’, balloon), expectant management and multiple combination treatments [1, 7, 8]. No particular treatment was considered optimal, likely due to the low methodological quality of the included studies, with few randomised controlled trials (RCTs) and predominantly case series and single‐centre cohort studies [1, 2, 6]. Furthermore, these reviews highlight the significant variation in outcomes reported and in the criteria used to define treatment success. It is this variation that leads to heterogeneity between studies and the inability to conduct a true comparison of treatments using quantitative data synthesis, and so uncertainty in how to manage CSEPs most effectively remains.

Studies assessing CSEP management generally focus on short term, post‐treatment outcomes, such as success or complications, but capturing future reproductive outcomes remains patchy. In view of this, the impact of intervention on future fertility is unclear. This makes counselling patients about their future fertility and risks challenging. Furthermore, the majority of studies focus on interventional treatments (medical or surgical modalities) with only a fraction assessing expectant management of failed or live CSEPs. A recent systematic review assessing only expectant management of CSEP included 492 patients but, as the included studies did not report the same outcomes, any conclusions drawn from that paper must be interpreted with caution [9].

We aim in this review to systematically describe the outcomes currently reported in the literature in studies investigating any type of CSEP treatment, including both termination by medical or surgical methods and continuation of the pregnancy. Our secondary aim is to evaluate the quality of outcome reporting.

## 2 Methods

This review is registered on the International prospective register of systematic reviews (PROSPERO; CRD42024508037) and has been conducted in accordance with the Preferred Reporting Items for Systematic Reviews and Meta‐Analyses (PRISMA) statement [10].

### 2.1 Literature Search

A comprehensive literature search was conducted in the electronic databases, PubMed, Medline and Google Scholar using the terms ‘Caesarean scar ectopic pregnancy' and 'Caesarean scar pregnancy’. The search covered the period between March 2014 and March 2024. Reference lists of all included articles and relevant reviews were also manually scanned for any relevant studies. The complete search strategy is provided in Table S1.

### 2.2 Study Selection

Two reviewers (S.N. and S.S.) independently screened titles, abstracts, and full text articles for eligibility. Any disagreements if not resolved by discussion were adjudicated by a third reviewer (C.B.). Inclusion criteria included studies of any design with a sample size of 50 or more women diagnosed with CSEP, where the primary aim of the study was to assess CSEP treatment, where a description of the mode of diagnosis (by ultrasound, MRI, surgery or histopathology) using standard diagnostic criteria was provided and the article was in the English language. Exclusion criteria included abstracts only, letters to the editor, reviews, studies where a treatment was not described and non‐English language articles.

Data extraction was performed independently by two reviewers (S.N. and S.S.) using a predefined data collection sheet. Study characteristics including first author, publication year, publishing journal, type of journal (general vs. specialist obstetrics and gynaecology), country of origin, study design, sample size, gestational age, method of diagnosis and type of intervention were extracted. Outcomes were documented as defined by the methods section of the papers, as primary or secondary. Where the outcome type was not clearly defined by the article, we considered the outcome equivalent to a secondary outcome for the purposes of quality assessment. To ensure our synthesis optimally reflected the maximum number of reported outcomes, we included outcomes if they were described either in the methods, results or discussion sections of the study, as some studies may have only mentioned certain outcomes in the discussion section but not the methods section of the paper. If outcome definitions were given for treatment success, they were recorded. Outcomes once extracted were classified into six pre‐defined domains according to the taxonomy principles recommended by COMET [11]: treatment sequelae; complications and adverse effects; morbidity and mortality; future reproductive health; obstetric outcomes of expectantly managed CSEP and quality of life.

### 2.3 Quality Assessment

Two reviewers (S.N. and S.S.) independently assessed each study's methodological quality using the Evidence Project risk of bias tool [12]. Eight items across three domains assessed study design, participant representativeness and equivalence of comparison groups. Reviewers rated each item as yes present, not present, and for some items not reported or not applicable.

Two reviewers (S.N. and S.S.) independently assessed the quality of outcome reporting and resolved any disagreements through discussion. A validated scoring criteria were used to assess the quality of outcome reporting, as described previously [13, 14, 15, 16]. The tool was modified to reflect that the majority of included studies did not have primary or secondary outcomes as defined by the study; we adapted secondary outcomes to also include outcomes that were not defined by the reviewers as primary or secondary. One point was given for each of the six domains: whether a primary outcome was clearly stated; whether the primary outcome was clearly defined for reproducible measures; whether the secondary outcomes were clearly stated; whether the secondary outcomes were clearly defined for reproducible measures; whether the authors explained the choice of outcome; and whether the methods that were used were appropriate to enhance the quality of measures. Pre‐defined cut‐offs of < 4 and 4 ≥ were used to categorise quality of outcome reporting into low‐ and high‐quality studies, as previously defined in the literature [13]. A point was awarded for stating a primary or secondary outcome, regardless of the total number of primary or secondary outcomes that were reported. Similarly, a point was awarded when at least one secondary outcome was clearly defined for reproducible measures, even if not all secondary outcomes were clearly described.

### 2.4 Data Analysis

Outcomes were classified into the previously described six major domains and the prevalence of outcomes across studies was reported as percentages. The association between the quality of outcome reporting and continuous variables: year of publication were assessed by nonparametric correlation coefficient (Spearman rho). Univariate analysis using the nonparametric Mann–Whitney *U* test was performed to measure the association between quality of outcome reporting and the type of journal (general vs. specialist obstetrics and gynaecology). We used a multivariate linear regression model to assess relationship associations between outcome reporting quality (the dependent variable) and the type of journal and the year of publication (as independent variables). Data analysis was performed using SPSS version 28.0.1.1 (IBM Corporation, Armonk, USA).

## 3 Results

### 3.1 Overview

**FIGURE 1:**
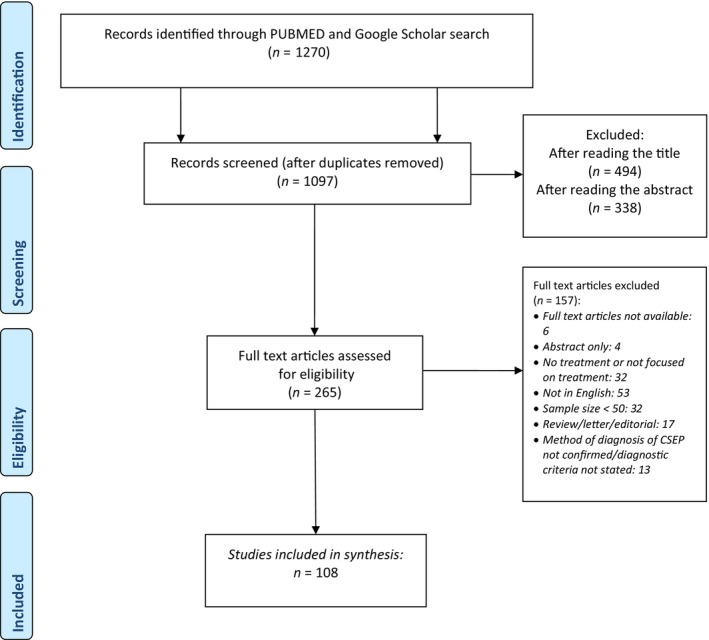
Flowchart showing studies identified through literature search (01.03.2014 to 05.03.2024).

A total of 1270 studies were identified through electronic searching. 1097 titles and abstracts were screened after removal of duplicate articles (Figure 1). We included 108 studies (Tables S2 and S3) in our analysis, reporting data on 17 941 women who underwent CSEP treatment. Trials consisted of 5 (5%) RCTs, 89 (82%) cohort studies and 14 (13%) case control studies. Of these, only 13% of studies were prospective in methodology.

**FIGURE 2:**
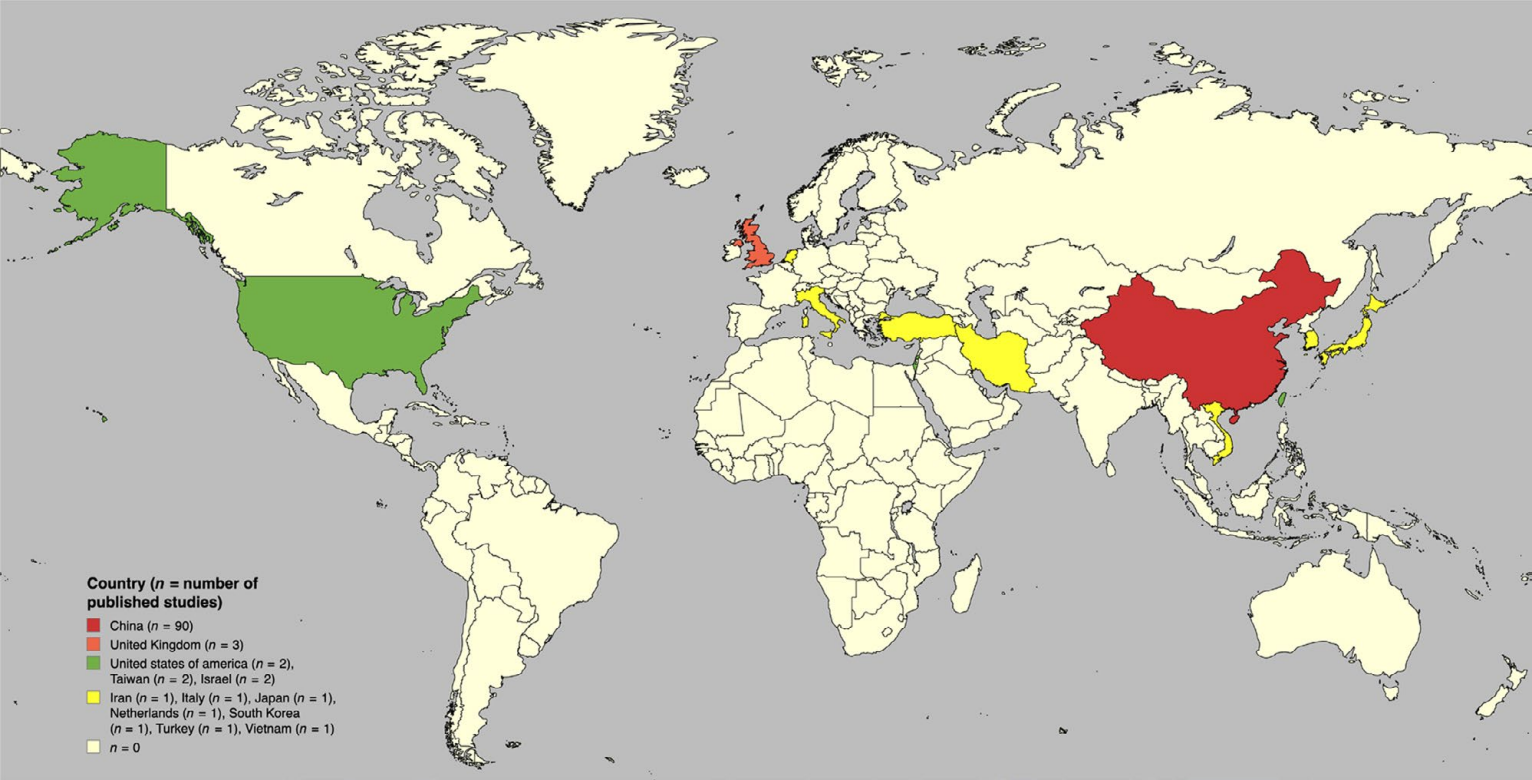
World map with an overview over the countries of origin for the included studies.

The large majority of studies (83%) originated from one country, China, with the remaining studies from the UK (3%), USA (2%), Israel (2%), Taiwan (2%) and the remaining being from the rest of the world (Figure 2). Sixty‐six (61%) studies were published in obstetrics and gynaecology‐specific journals and 42 (39%) studies were published in general medical journals. The 108 included studies had a median sample size of 102 participants (range 50–1373). All cases were diagnosed with ultrasound imaging, with 31 (29%) studies using MRI as an additional diagnostic tool to ultrasound. Gestation at treatment varied, with 10 (9%) studies not actually specifying the gestational age of participants. In the remaining studies, 74 (69%) women were treated in the first trimester, 19 (18%) in the first and second trimester and 5 (5%) included women in all three trimesters.

The included studies reported a wide range of aims from assessing treatment efficacy, safety, success rate, effect on serum human chorionic gonadotropin (hCG) levels, complication rate, risk factors associated with bleeding, pain and failed treatment, optimal time interval between UAE and other treatments, effect of gestational age and type of CSEP on outcomes, subsequent fertility outcomes, and evaluating the natural history of CSEP.

Eight different management options were included, alone or in combination: expectant management of live or failed pregnancy, medical management (with local or systemic methotrexate), surgical management (suction curettage or resection via multiple routes—vaginal, hysteroscopic, laparoscopic, abdominal, hysterectomy), UAE, HIFU, local sclerotherapy, abdominal aortic balloon occlusion and uterine balloon insertion. Included studies evaluated either one treatment option or included several arms comparing different types of treatment; the most commonly used treatments were surgical management in 101 (94%) studies, followed by medical management in 62 (57%) studies and expectant management in only 9 (8%) studies.

The five included RCTs were from two countries, China (80%) and Italy (20%) with a median sample size of 101 participants (range 54–144) (Table S3). In four studies (80%) participants were treated in the first trimester [17, 18, 19, 20], however, in one study [21] no gestation was specified. Treatments varied from UAE followed by dilatation and curettage (D&C) at different time intervals [17]; local MTX followed by hysteroscopic resection versus ultrasound guided D&C [18]; UAE followed by D&C versus UAE followed by hysteroscopy and curettage [20]; local versus systemic MTX [19]; and UACE followed by D&C with hysteroscopy monitoring versus ultrasonography monitoring versus no monitoring [21]. The primary aims of these studies included evaluating the optimal time interval between different treatment modalities, comparing success rates of different treatments, assessing treatment safety and complication rates. Only two studies defined and reported a primary outcome: success rate [18] versus number of participants with short‐term complications 2 months following D&C [21] Successful treatment was defined and reported in two studies as no further treatment required until complete resolution of the CSEP based on decline of serum hCG levels and the absence of residual pregnancy tissue [18] versus resolution of ultrasonographic findings and normalisation of serum hCG levels within 60 days [19].

### 3.2 Outcomes

Across 108 studies, we identified a total of 326 outcomes. By eliminating similar outcomes, we consolidated these into 88 clinically relevant final outcomes, which were then categorised into six domains (Table 1). The most common reported outcomes were total blood loss (93 of 108 trials, 86%), additional interventions after the planned primary treatment (83 of 108 trials, 77%), and the time for serum hCG to normalise post‐treatment (75 of 108 trials, 69%). The most commonly reported complication was major haemorrhage. Quality of life outcomes were reported in one trial using the World Health Organisation Quality of Life—BREF measure, which evaluated physical health, psychological well‐being, environmental health and social relationships [22]. The most prevalent domains were treatment‐related sequelae (98 of 326 outcomes, 30%), complications and adverse events (94 of 326 outcomes, 29%) and future reproductive health outcomes (93 of 326 outcomes, 29%). The median quality score was 3 of 6 (interquartile range 3–4) (Table S2). Scores of 2, 3 and 4 accounted for 89% of the studies. A primary outcome was defined and reported in 11 (10%) studies, and success of treatment was reported in 71 (66%) studies, but the criteria for success were not clearly defined in 21 (30%) of them.

**TABLE 1:** Outcome reporting in CSEP trials: outcomes reported by domain (*n* = 88).

Outcome domain	Outcome	Studies, *n* (%)
Morbidity and mortality	Sepsis	1 (< 1)
	Haemorrhagic shock	5 (5)
	DIC	3 (3)
	Renal failure	1 (< 1)
	Admission to ICU	1 (< 1)
	Death	6 (6)
Treatment sequelae^a^	Success of treatment	71 (66)
	Failure of treatment	30 (28)
	Total blood loss	93 (86)
	Blood transfusion	32 (30)
	Additional intervention after the planned primary treatment	83 (77)
	Hysterectomy	60 (56)
	Procedure time	41 (38)
	Total duration of hospital stay	68 (63)
	Total hospitalisation cost	34 (31)
	Readmission rate	14 (13)
	Haematoma in caesarean scar (where not an intentional effect in evacuation with cervical suture)	2 (2)
	Time for serum hCG to normalise post treatment	75 (69)
	Time for RPOC resolution	31 (29)
	Length of time of recovery	11 (10)
Complications/Adverse events of treatment	Complication/side effect rate	40 (37)
	Major haemorrhage	64 (59)
	Conversion to laparoscopy	2 (2)
	Conversion to laparotomy	7 (6)
	Damage to surrounding structures	30 (28)
	Uterine rupture	1 (< 1)
	Uterine perforation	30 (28)
	Retained pregnancy tissue	45 (42)
	Development of AMV or EMV following treatment	3 (3)
	Post operative pain	41 (38)
	Fever	27 (25)
	Infection/inflammation post treatment	22 (20)
	Postop embolisation syndrome^b^	3 (3)
	Methotrexate toxicity	2 (2)
	Anomalous renal/hepatic/coagulation function/FBC	13 (12)
	Myelosuppression	5 (5)

	Embolism(arterial)/thrombosis	5 (5)
	Amniotic embolism	2 (2)
	Cardiopulmonary adverse reactions	5 (5)
	Gastrointestinal adverse reactions	22 (20)
Anaesthetic related side effects (headache/urinary retention)	1 (< 1)
Obstetric (maternal and foetal) outcomes of expectantly managed CSEP	Pregnancy loss pre‐viability	1 (< 1)
	Hysterotomy	1 (< 1)
	Cervical insufficiency	1 (< 1)
	Preterm labour	1 (< 1)
	Medical complications of pregnancy	1 (< 1)
	Severity of bleeding antepartum	1 (< 1)
	Severity of bleeding postpartum	2 (2)
	Uterine dehiscence	1 (< 1)
	Uterine rupture	4 (4)
	Abnormal placentation	6 (6)
	Live birth	4 (4)
	Gestational age at delivery	8 (7)
	Mode of delivery	7 (6)
	Caesarean without hysterectomy	2 (2)
	Elective caesarean hysterectomy	2 (2)
	Emergency caesarean hysterectomy	5 (5)
	Blood loss at delivery	2 (2)
	Blood transfusion	2 (2)
	Neonatal outcomes	2 (2)
	Additional intervention required (medical/surgical treatment of initially expectantly managed CSEP)	2 (2)
Future reproductive health following resolution of index CSEP	Time for menstruation to return to normal	37 (34)
	Prolonged amenorrhoea	21 (19)
	Abnormal menstruation	19 (18)
	Intrauterine adhesions	11 (10)
	Caesarean scar niche condition at follow‐up	10 (9)
	RMT post treatment at follow‐up	4 (4)
	Thickness of endometrium on follow‐up	2 (2)
	Ovarian function	9 (8)
	Desire for future fertility	17 (16)
	Subfertility	12 (11)
	Interval between CSEP treatment and subsequent pregnancy	9 (8)

	Subsequent pregnancy rate	34 (31)
	Mode of subsequent conception	6 (6)
	Normal subsequent pregnancy rate	8 (7)
	Successful subsequent pregnancy	12 (11)
	Recurrent CSEP rate	28 (26)
	Type of CSEP recurrence	1 (< 1)
	Subsequent non CSEP ectopic	10 (9)
	Miscarriage rate in subsequent pregnancy	18 (17)
	Termination of pregnancy rate in subsequent pregnancy	20 (19)
	Subsequent pregnancy related complications	11 (10)
	Abnormal placentation in subsequent pregnancy	14 (13)
	Uterine rupture in subsequent pregnancy	1 (< 1)
	Gestational age at delivery in subsequent pregnancy	23 (21)
	Mode of delivery in subsequent pregnancy	17 (16)
Neonatal outcome in subsequent pregnancy	2 (2)
Quality of life	Quality of life	1 (< 1)

Abbreviations: AMV, arterial vascular malformation; CSEP, caesarean scar ectopic pregnancy; DIC, disseminated intravascular coagulation; EMV, enhanced myometrial vascularity; FBC, full blood count; hCG, human chorionic gonadotropin; ICU, intensive Care Unit; RMT, Residual myometrial thickness; RPOC, retained products of conception.

^a^ Where applicable to that specific treatment, covers all treatment modalities except expectant management.

^b^ Symptoms include pelvic/abdominal/hip pain, nausea, vomiting, fever and perineal swelling.

We explored the relationship between the quality of outcome reporting and the year of publication and type of journal (Table 2). Univariate and multivariate analysis showed that neither of these factors were significantly associated with quality of outcome reporting.

**TABLE 2:** Outcome reporting in CSEP trials: multiple linear regression analyses to determine factors associated with quality of outcome reporting.

Factor	Univariable		Multivariable	
	Rho Spearman	*p*	*β*	*p*
Journal type (specialist/generalist)^a^	—	0.503	0.125	0.515
Year of publication	0.152	0.116	0.041	0.192

^a^ Based on Mann–Whitney *U* test.

We completed risk of bias assessment using ‘The Evidence Project risk of bias tool’. This tool does not provide a summary score, or judgement and we have therefore presented the results of the items as a simple checklist (Table S4). Generally, the common reasons for bias were the retrospective methodology of the studies and the lack of random selection of participants for assessment.

## 4 Discussion

### 4.1 Main Findings

In this review, we identified substantial heterogeneity in outcome reporting with over 300 outcomes reported across 108 studies. The majority of studies were from one country, China. The most common reported outcomes were total blood loss, additional intervention after the planned primary treatment, and time for serum hCG to normalise post‐treatment. The primary outcome was only reported in a small handful of studies. No relationship was identified between quality of outcome reporting and year of study publication and type of journal.

### 4.2 Interpretation

This review presents outcomes that have been deemed important by researchers and healthcare professionals, with only one study reporting outcomes related to quality of life and no studies focusing on patient‐centred outcomes such as patient satisfaction or psychological sequelae of the treatment. A recent review of outcome reporting in ‘ectopic pregnancies’ also identified that only a minority of studies focused on psychological impact (3%) or treatment satisfaction (13%) [23, 24].

Outcomes of expectantly managed live CSEPs are very important as women may consider continuing with a CSEP and have a right to be counselled effectively about the implications in terms of potential maternal and foetal outcomes and their future fertility. Unfortunately, reporting on these outcomes is sporadic across studies, which is to some extent understandable bearing in mind anecdotal evidence showing serious adverse outcomes in cases of CSEP which progressed beyond the second trimester. This has also influenced the recent Society for Maternal‐Fetal Medicine guidelines to ‘recommend against expectant management of caesarean scar ectopic pregnancy’ [25]. This judgement is not based on high‐quality evidence and does not take into account individual circumstances such as the exact implantation site and extension into the cervical canal.

Most included studies originated from China, likely due to the country's high CD rates following the shift from the one‐child to two‐child policy [26]. Consequently, many of these women will have had their first child via CD during the one‐child policy, which increases the risk of CSEP in subsequent pregnancies.

The lack of association between quality of outcome reporting and year of study publication and type of journal suggests that the emphasis has been placed on the results rather than on the quality of outcome reporting. This approach increases the risk of bias resulting in overestimation of the efficacy of various treatments. The lack of high‐quality RCTs (5/108) further adds to the uncertainty regarding the effectiveness and safety of treatments for CSEP. Poor quality studies perpetuate research waste, as important outcomes are not assessed and reported or interpreted in the wider context of what has already been published [27]. This diminishes the quality of patient counselling and care.

### 4.3 Strengths and Limitations

To the best of our knowledge, this is the first and largest systematic review to describe variation in outcome reporting in CSEP treatment studies. A robust, original methodology was employed with the use of two independent reviewers to assess and select studies to prevent bias. All study designs from observational studies to RCTs were included to minimise the risk of missing any outcomes and to accurately reflect current outcome reporting. We limited our review to a 10‐year period because before 2014, there were relatively few CSEP studies with small sample sizes and limited treatment options. This is evident from the 1538 articles published from the inception of the PubMed database until 2013, compared to 1952 articles published just between 2014 and 2024, highlighting not only the proliferation of treatment combinations now available but also the publication of larger studies.

However, including a range of different methodological study designs limited our ability to compare study quality. ‘The Evidence Project risk of bias tool’ was therefore used which covers all study designs but does not provide a summary score that can be interpreted, putting the burden of interpretation of study risk of bias from the reviewer to the reader [28]. The type and quality of outcome reporting is likely to have been influenced by bias generated by the retrospective nature of the majority of studies. RCTs are considered to be the gold standard tool to provide scientific evidence [29] but limiting our review to this study design only would have curtailed our ability to report accurately on outcomes, given we only identified five RCTs. We also excluded articles written in languages other than English and given the number of articles from Asia we inevitably will have excluded a number of potentially relevant studies.

## 5 Conclusion

This review highlights a wide variation in outcome reporting and the lack of patient‐centred outcomes in studies on CSEP treatment, showing that there is an urgent need for better standardisation of research in this area.

We propose development of an international consensus with key stakeholders, including people with a lived experience of CSEP, to select outcomes which are relevant to all. This review is the first in a series of steps to determine which outcomes should be prioritised in future research in CSEP treatment. The International Collaboration—Core outcomes for caesarean scar ectopic pregnancy research (COSCAR) aims to develop a core outcome set for research in CSEP treatment. The Core Outcomes in Women's Health initiative supported by journal editors encourages publication of studies using core outcome sets, as this will lead to reduction in bias due to selective outcome reporting and provide comparable data which will ultimately lead to improved evidence‐based patient care [30]. Given the global increase in CDs [31] and the predicted associated increase in incidence of CSEP we urgently need high‐quality research to inform patient counselling and management.

## Author Contributions

The conception and design of this paper were conceived by the COSCAR steering committee (S.N., I.E.T.‐T., A.K.A., J.L., K.Y.C., M.O., R.A., F.D.A., L.N.V., B.M., C.B. and D.J.). S.N. and S.S. conducted the literature search and primary analysis with review from C.B. S.N. wrote the initial draft manuscript. All authors contributed to interpretation of the data, revised this article critically, and agreed upon the final manuscript prior to submission.

## Conflicts of Interest

The authors declare no conflicts of interest.

## Supporting information


Data S1.

## Data Availability

The data that support the findings of this study are available from the corresponding author upon reasonable request.
