# Supplementary material for: Outcome Reporting in Studies Investigating Treatment for Caesarean Scar Ectopic Pregnancy: A Systematic Review
Source: BJOG. 2024 Nov 7;132(3):278–87. doi: 10.1111/1471-0528.17989 (PMC11704075; doi:10.1111/1471-0528.17989)
Supplement: Supplementary file 1 — Data S1. [file BJO-132-278-s001.docx]

**Appendix Table S1 Outcome reporting in CSEP trials: Search strategy**

| **Search PubMed** | **Query** |
| --- | --- |
| #3 | #1 AND #2 |
| #2 | "Ultrasonography"[tiab] OR ultras* [tiab] OR sonohysterogra*[tiab] OR sonogra*[tiab] OR hysterosonogra*[tiab] OR echogra*[tiab] OR “transvaginal”[tiab] OR “transabdominal”[tiab] OR “three dimensional”[tiab] OR “3D”[tiab] OR “MRI”[tiab] OR “Magnetic resonance imaging”[tiab] OR “power Doppler”[tiab] OR “colour Doppler”[tiab] “color Doppler”[tiab] OR “pulsed Doppler”[tiab] OR “Doppler”[tiab] OR diagnos*[tiab] OR “definition” [tiab] OR classifi*[tiab] OR “hysteroscopy”[tiab] |
| #1 | “cesarean scar pregnancy”[tiab] OR “caesarean scar pregnancy”[tiab] OR "caesarean scar ectopic pregnancy"[tiab] OR "cesarean scar ectopic pregnancy"[tiab] OR “caesarean scar implantation"[tiab] OR “cesarean scar implantation"[tiab] OR "cesarean section scar ectopic pregnancy"[tiab] OR "caesarean section scar pregnancy"[tiab] OR "cesarean section scar pregnancy"[tiab] OR "cesarean ectopic pregnancy"[tiab] OR “intrauterine ectopic”[tiab] OR cicatrix pregnancy[tiab] OR isthmocele pregnancy[tiab] OR niche pregnancy[tiab] OR caesarean scar defect pregnancy[tiab] OR cesarean scar defect pregnancy[tiab] OR cesarean scar diverticulum pregnancy[tiab] OR caesarean scar diverticulum pregnancy[tiab] OR caesarean ectopic[tiab] OR cesarean ectopic[tiab] |
| **Search Google Scholar** | **Query** |
| #1 | *allintitle: (cesarean OR caesarean) AND scar pregnancy OR ectopic OR ultrasonography OR ultrasound OR sonography OR transvaginal OR transabdominal OR MRI OR three-dimensional OR Doppler OR diagnosis OR definition OR classification OR hysteroscopy* |

**Table S2 Outcome reporting in CSEP trials: Study characteristics (n=103)**

| **Author, year** | **Journal**  **(1=specialist,**  **2=general)** | | **Country** | | **Study design** | | **Sample size** | | **Intervention(s)** | | **Outcome quality score** | | **Definition of successful treatment** | |
| --- | --- | --- | --- | --- | --- | --- | --- | --- | --- | --- | --- | --- | --- | --- |
| **Prospective case-control studies** | | | | | | | | | | | | | | |
| **Tarafdari 2024^1^** | **1** | | Iran | | prospective case-control | | 67 | | Systemic MTX +/- fetal reduction vs double-balloon cervical ripening catheter inserted under transabdominal ultrasound guidance | | 4 | | ND | |
| **Retrospective case-control studies** | | | | | | | | | | | | | | |
| **Fu 2023^2^** | **1** | | China | | retrospective case-control | | 98 | | Transabdominal ultrasound-guided hysteroscopic curettage | | 3 | | NR | |
| **Cao 2022^3^** | **2** | | China | | retrospective case-control | | 80 | | UAE + D&C vs UAE + hysteroscopy + curettage | | 4 | | ND | |
| **Liu Xu 2022^4^** | **1** | | China | | retrospective case-control | | 60 | | Ultrasound-guided curettage combined with hysteroscopic electro-resection after injection of pituitrin vs hysteroscopic electro-resection after MTX chemotherapy | | 4 | | No complications or serious adverse reactions are observed, and postoperative hCG decreases significantly. The ultrasound re-examination shows that scar pregnancies have been completely removed, and hysterectomy is not required. | |
| **Wang 2022^5^** | **1** | | China | | retrospective case-control | | 181 | | Hysteroscopic or laparoscopic operation +/- preventive UAE before | | 5 | | NR | |
| **Zhu 2022^6^** | **2** | | China | | retrospective case-control | | 142 | | Systemic MTX combined with B ultrasound-monitored clearance vs MTX + UAE+B ultrasound-monitored clearance | | 4 | | hCG decreased to normal, vaginal bleeding decreased or stopped, abdominal pain disappeared, and US showed a smaller pelvic mass | |
| **Liu 2021^7^** | **1** | | China | | retrospective case-control | | 55 | | Suction curettage under ultrasound guidance or laparoscopic surveillance vs scar pregnancy lesion resection by laparoscopy or open surgery vs systemic MTX | | 2 | | NR | |
| **Liu 2020^8^** | **1** | | China | | retrospective case-control | | 225 | | Suction curettage under hysteroscopic guidance +/- pre HIFU treatment | | 3 | | Patients recovered without secondary treatment | |
| **Lu 2020^9^** | **1** | | China | | retrospective case-control | | 70 | | MTX (systemic/local) + uterine curettage under hysteroscopic guidance and ultrasound monitoring +/- Foley balloon catheter +/- UAE prior to surgery | | 3 | | NR | |
| **Xiao 2019^10^** | **2** | | China | | retrospective case-control | | 103 | | Local or systemic MTX injection and surgery (D&C, laparoscopy and/or hysteroscopy) vs UAE and surgery vs surgery only | | 3 | | NR | |
| **Wang 2018^11^** | **2** | | China | | retrospective case-control | | 80 | | Ectopic Pregnancy II and MTX pretreatment combined with hysteroscopic electrosurgical treatment | | 2 | | NR | |
| **Gui 2017^12^** | **2** | | China | | retrospective case-control | | 120 | | Suction curettage under the surveillance of ultrasound with or without additional interventions | | 3 | | NR | |
| **Zhang 2017^13^** | **1** | | China | | retrospective case-control | | 331 | | Suction curettage (including ‘direct’ curettage or curettage following MTX, UAE + curettage) vs surgical resection (including abdominal, vaginal, or laparoscopic route) vs hysteroscopic excision vs hysterectomy | | 2 | | NR | |
| **Wang 2015^14^** | **1** | | China | | retrospective case-control | | 458 | | D&C under ultrasound guidance +/ -adjunctive UAE | | 5 | | NR | |
| **Prospective cohort studies** | | | | | | | | | | | | | | |
| **Sun 2023^15^** | **2** | | China | | prospective cohort | | 64 | | Curettage and hysteroscopy after contrast-enhanced ultrasound-guided sclerotherapy with lauromacrogol injection or UAE or UAE + MTX pretreatment | | 4 | | ND | |
| **Wu Guo 2023^16^** | **1** | | China | | prospective cohort | | 68 | | Prophylactic abdominal aortic balloon occlusion OR UAE before laparoscopic removal of gestational tissues and repair of uterine caesarean scar defects | | 4 | | ND | |
| **Tan 2022^17^** | **2** | | China | | prospective cohort | | 77 | | Local intra-gestational sac MTX injection + D&C under US guidance +/- Foley balloon catheter vs UAE (MTX + gelatin) + D&C +/- Foley balloon catheter | | 6 | | Normalisation of ultrasonographic findings and hCG, no uterine rupture, no conversion to surgical resection, blood loss $\leq$500mL during operation and follow up, and no required blood transfusion | |
| **Sun 2021^18^** | **2** | | China | | prospective cohort | | 53 | | Uterine curettage +/- hysteroscopy or laparoscopy or transvaginal resection combined by hysteroscopy | | 4 | | NR | |
| **Ou 2020^19^** | **1** | | China | | prospective cohort | | 105 | | Suction curettage +/- UAE | | 4 | | Complete recovery with no adjuvant treatments or severe adverse events (blood loss>500ml or hysterectomy). Complete recovery meant that hCG had decreased to normal or that the mass in the uterus had disappeared within 60 days after treatment. | |
| **Harb 2018^20^** | **1** | | UK | | prospective cohort | | 92 | | Expectant management vs ultrasound guided D&C vs laparoscopy/laparotomy/laparoscopy + hysteroscopy + balloon + UAE | | 5 | | Complete resolution of pregnancy without the need for further intervention following primary management | |
| **Timor-Tritsch 2015^21^** | **2** | | USA | | prospective cohort | | 60 | | Expectant management vs local MTX vs Foley balloon catheter vs D&C vs U+E | | 2 | | NR | |
| **Wu 2014^22^** | **2** | | China | | prospective cohort | | 61 | | Intra-arterial MTX+ UAE+ laparoscopy + hysteroscopy + repair of defect vs. intra-arterial MTX+ UAE + uterine curettage | | 3 | | ND | |
| **Retrospective cohort studies** | | | | | | | | | | | | | | |
| **Ban 2023^23^** | **1** | | China | | retrospective cohort | | 955 | | Suction curettage, hysteroscopy, laparoscopy or laparotomy after pretreatment with MTX, mifepristone, or UAE | | 6 | | Disappearance of the gestational sac and normalisation of serum hCG | |
| **Cagli 2023^24^** | **1** | | Turkey | | retrospective cohort | | 56 | | Contents of the sac were aspirated followed by transvaginal ultrasound-guided single-dose local MTX | | 3 | | Disappearance of the gestational sac and normalisation of serum hCG | |
| **Huo 2023^25^** | **1** | | China | | retrospective cohort | | 725 | | Systemic MTX vs local and systemic MTX vs D&C vs UAE + D&C vs hysteroscopic curettage vs laparoscopy + hysteroscopic curettage | | 3 | | Return of hCG to normal without further surgical treatment | |
| **Kaelin Agten 2023^26^** | **1** | | Global | | retrospective cohort | | 460 | | Surgical management vs medical management vs balloon catheter vs other rarer management options | | 4 | | CSP resolved fully with no need for any additional medical or surgical intervention | |
| **Lu 2023^27^** | **1** | | China | | retrospective cohort | | 131 | | Hysteroscopic surgery + electric suction device under abdominal ultrasound guidance +/- pre-ultrasound-guided local injection of lauromacrogol | | 6 | | hCG decreased to normal and the anterior isthmus mass disappeared | |
| **Meyer 2023^28^** | **1** | | NS | | retrospective cohort | | 55 | | IM MTX vs IM MTX + ultrasound-guided needle aspiration vs surgery vs continuing the pregnancy | | 3 | | NR | |
| **Mu 2023^29^** | **1** | | China | | retrospective cohort | | 127 | | Systemic MTX combined with suction curettage | | 3 | | No need for additional intervention after treatment and recovery of menstruation and normalisation of hCG | |
| **Shen 2023^30^** | **1** | | China | | retrospective cohort | | 181 | | Ultrasound-guided vacuum aspiration +/- after UAE +/- after local injection of lauromacrogol +/- without any pretreatment | | 4 | | Complete recovery without severe complications (blood loss of ≥ 200 mL and uterine rupture) and without additional therapy | |
| **Shiber 2023^31^** | **1** | | Israel | | retrospective cohort | | 60 | | Expectant vs systemic+/- local MTX vs suction curettage vs hysteroscopy vs wedge resection by laparoscopy or laparotomy | | 2 | | NR | |
| **Spong 2023^32^** | **1** | | USA | | retrospective cohort | | 101 | | Expectant management | | 3 | | NR | |
| **Van 2023^33^** | **1** | | Vietnam | | retrospective cohort | | 123 | | Combined systemic and local MTX injection | | 4 | | Normalisation of hCG (<5 UI) and regression of US findings without surgical intervention | |
| **Verberkt 2023^34^** | **1** | | Netherlands | | retrospective cohort | | 60 | | Expectant management vs MTX vs curettage with temporary cervical cerclage vs laparoscopic niche resection | | 5 | | Uneventful decline in hCG and resolution of pregnancy tissue without the need for additional treatment | |
| **Wang 2023^35^** | **1** | | China | | retrospective cohort | | 272 | | HIFU or UAE+ US guided D&C | | 4 | | Treatment completed as planned, with no major bleeding (≥ 100 ml), hCG returned to normal, the menstruation cycle returned to normal, no need for other drug treatment (including MTX and mifepristone) or further surgical intervention and no significant complications | |
| **Wu 2023^36^** | **1** | | China | | retrospective cohort | | 100 | | Ultrasound-guided suction curettage vs hysteroscopic resection | | 4 | | Complete resolution of CSP and hCG (<5 mIU/mL) with no major complications (massive bleeding and perforation of uterus) and no need for additional treatment | |
| **Yang 2023^37^** | **2** | | China | | retrospective cohort | | 223 | | Ultrasound-guided vacuum aspiration followed by supplementary curettage +/- adjuvant systemic MTX vs UAE vs hysteroscopy | | 4 | | ND | |
| **Yang Cao 2023^38^** | **1** | | China | | retrospective cohort | | 1373 | | Ultrasound-guided evacuation vs ultrasound-guided evacuation with UAE pretreatment vs hysteroscopy-guided evacuation vs hysteroscopy-guided evacuation with UAE pretreatment | | 4 | | ND | |
| **Zeng 2023^39^** | **1** | | China | | retrospective cohort | | 314 | | Curettage + pituitrin combined with ultrasonic monitoring and hysteroscopy-guided surgery vs curettage after local MTX vs laparoscopic or transvaginal or transabdominal resection | | 3 | | ND | |
| **Chen 2022^40^** | **1** | | Taiwan | | retrospective cohort | | 53 | | UAE followed by D&C | | 2 | | ND | |
| **Fu 2022^41^** | **2** | | China | | retrospective cohort | | 278 | | Laparoscopy vs laparotomy +/- pre-treatment with UAE or (temporary/permanent) arterial occlusion | | 2 | | NR | |
| **Gu 2022^42^** | **2** | | China | | retrospective cohort | | 54 | | UAE combined with D&C using ultrasound | | 3 | ND | | |
| **Hong 2022^43^** | **2** | | China | | retrospective cohort | | 326 | | Ultrasound-guided local injection of MTX vs UAE combined with suction aspiration vs ultrasound-guided suction aspiration vs ultrasound-guided local injection of lauromacrogol combined with suction aspiration | | 3 | No remnants on US and hCG decreased to normal (<5 IU/L) | | |
| **Liu 2022^44^** | **1** | | China | | retrospective cohort | | 153 | | HIFU + US guided D&C | | 3 | Efficiency of first line treatment | | |
| **Peng 2022^45^** | **2** | | China | | retrospective cohort | | 153 | | HIFU+US guided suction curettage | | 4 | Successful removal of pregnancy tissue and a back-to-normal hCG with no need for additional UAE or surgery treatment | | |
| **Qu 2022^46^** | **1** | | China | | retrospective cohort | | 447 | | MTX + surgery vs ultrasound-guided curettage directly vs curettage combined with hysteroscopy | | 4 | No additional treatment needed, no retained mass of conception and hCG returned to a normal within 4 weeks | | |
| **Shao 2022^47^** | **1** | | China | | retrospective cohort | | 276 | | Direct hysteroscopy vs UAE + hysteroscopy vs systemic MTX prior to hysteroscopy | | 4 | Complete recovery with no need for additional interventional treatment, and the absence of severe complications | | |
| **Xu 2022^48^** | **1** | | China | | retrospective cohort | | 1126 | | Hysteroscopic lesion excision vs US guided curettage vs laparoscopic lesion excision vs transabdominal lesion excision vs transvaginal lesion excision + adjuvant UAE | | 3 | | NR | |
| **Zhou 2022^49^** | **1** | | China | | retrospective cohort | | 160 | | Ultrasound-guided vacuum aspiration after local injection of lauromacrogol vs ultrasound-guided vacuum aspiration after UAE vs transabdominal resection or hysteroscopy combined with laparoscopic resection | | 3 | | Complete recovery without severe complications (such as uterine rupture and massive bleeding), without additional treatment | |
| **Cao 2021^50^** | **2** | | China | | retrospective cohort | | 87 | | UAE + local MTX + hysteroscopy + curettage vs transvaginal removal and repair | | 3 | | Complete recovery without severe complications such as heavy vaginal bleeding (>200ml), gastrointestinal perforation, and uterine rupture without second-line therapy | |
| **Chen 2021^51^** | **2** | | China | | retrospective cohort | | 83 | | Laparoscopy combined with hysteroscopy +/- temporary Ligation of the Bilateral Uterine Arteries | | 4 | | ND | |
| **De Braud 2021^52^** | **1** | | UK | | retrospective cohort | | 62 | | Suction curettage +/- Shirodkar cervical suture | | 6 | | NR | |
| **Lan 2021^53^** | 2 | | China | | retrospective cohort | | 98 | | UAE (MTX+gelatin sponge) + D&C | | 4 | | NR | |
| **Levin 2021^54^** | **1** | | Israel | | retrospective cohort | | 63 | | Single- vs multiple-dose MTX | | 5 | | NR | |
| **Lin 2021^55^** | **2** | | China | | retrospective cohort | | 55 | | Transvaginal curettage after UAE vs transabdominal ultrasound-guided hysteroscopic curettage vs laparoscopic cesarean scar resection | | 4 | | NR | |
| **Mitsui 2021^56^** | **2** | | Japan | | retrospective cohort | | 57 | | Elective TAH vs D&C vs UAE+D&C vs Transcervical resection vs MTX +KCL vs continuing the pregnancy | | 1 | | NR | |
| **Shen 2021^57^** | **2** | | China | | retrospective cohort | | 71 | | Systemic/local MTX+/-curettage vs UAE + curettage vs hysteroscopy + laparoscopy | | 3 | | Decline of hCG, the absence of the GS on US after treatment and no additional treatment required | |
| **Tang 2021^58^** | **1** | | China | | retrospective cohort | | 439 | | Hysteroscopy combined with D&C vs systemic MTX followed by hysteroscopy combined with D&C vs UAE or laparoscopic ligation of bilateral uterine arteries followed by hysteroscopy combined with D&C | | 3 | | Sufficiently decreased hCG and no additional treatment required | |
| **Wu 2021^59^** | **1** | | China | | retrospective cohort | | 135 | | Transvaginal resection vs laparoscopic resection vs UAE+ hysteroscopic curettage vs UAE + uterine curettage vs hysteroscopic curettage | | 2 | | NR | |
| **Xiong 2021^60^** | **1** | | China | | retrospective cohort | | 154 | | UAE + uterine curettage vs MTX + uterine curettage vs uterine curettage alone | | 3 | | Absence of residual uterine pregnancy tissue, hCG returns to normal, and menstruation resumes | |
| **Xu 2021^61^** | **1** | | China | | retrospective cohort | | 117 | | Ultrasound-guided curettage vs laparoscopy-monitored curettage vs laparoscopic resection +/- pre-UAE | | 3 | | NR | |
| **Yuan 2021^62^** | **1** | | China | | retrospective cohort | | 52 | | HIFU combined with ultrasound-guided suction curettage | | 4 | | NR | |
| **Cheng 2020^63^** | **1** | | China | | retrospective cohort | | 131 | | Laparoscopy assisted by operative hysteroscopy (LAOH) vs UAE followed by LAOH vs ultrasound‑guided D&C vs UAE followed by D&C | | 3 | | Continuous decrease of serum hCG and disappearance of GS, with no need for additional treatment or change of surgical methods | |
| **Fang 2020^64^** | **2** | | China | | retrospective cohort | | 154 | | Laparoscopy vs hysteroscopy vs hysteroscopy-laparoscopy vs UAE + curettage vs HIFU + curettage | | 4 | | Did not require other complementary therapy | |
| **Huang Li 2020^65^** | **1** | | China | | retrospective cohort | | 257 | | Direct suction curettage + Foley ballon vs preprocessing (with MTX, oral mifepristone, MTX + mifepristone, UAE/UACE) or suction curettage + Foley ballon | | 3 | | Removal of the pregnancy with subsequent negative hCG and confirmed chorionic villi on pathological examination | |
| **Huang Zhao 2020^66^** | **2** | | China | | retrospective cohort | | 173 | | Hysteroscopy and laparoscopy vs UAE+MTX + hysteroscopy vs UAE+ curettage | | 3 | | NR | |
| **Tan Sun 2020^67^** | **2** | | China | | retrospective cohort | | 183 | | Evacuation vs UAE vs laparoscopic surgery | | 3 | | ND | |
| **Wu 2020^68^** | **2** | | China | | retrospective cohort | | 151 | | USG-LLI or UAE + MTX + curettage followed by hysteroscopy | | 4 | | Complete recovery without severe complications (such as massive bleeding, gastrointestinal perforation and uterine rupture), without second line therapy | |
| **Yin 2020^69^** | **1** | | China | | retrospective cohort | | 69 | | Mifepristone or MTX + curettage vs UAE + curettage vs laparotomy | | 3 | | Reduction or disappearance of the mass and/or significant normalisation of hCG | |
| **Zhang 2020^70^** | **2** | | China | | retrospective cohort | | 112 | | Ultrasound-guided curettage and hysteroscopy without repair of the defect vs ultrasound-guided curettage and hysteroscopy and repair of the defect under laparoscopy | | 3 | | ND | |
| **Fei 2019^71^** | **2** | | China | | retrospective cohort | | 204 | | Transvaginal clearance vs endoscopic surgery (laparoscopy + defect repair +/- hysteroscopy +/- pre MTX +/- mifepristone) vs UAE+MTX | | 3 | | NR | |
| **Le 2019^72^** | **1** | | China | | retrospective cohort | | 313 | | D&C under ultrasound guidance vs D&C with hysteroscopic guidance vs vaginal excision vs laparotomy vs laparoscopy | | 3 | | hCG decreased >15% a week after the procedure | |
| **Qiu 2019^73^** | **2** | | China | | retrospective cohort | | 62 | | UAE combined with D&C guided by ultrasonography or hysteroscopy | | 4 | | Disappearance of the CSP sac and normalised hCG without an additional intervention requirement or any severe complication, such as bleeding, uterine rupture, or hysterectomy | |
| **Qiuyang 2019^74^** | **1** | | China | | retrospective cohort | | 101 | | Ultrasound-guided local injection of MTX + oral mifepristone | | 3 | | hCG decreased to normal, and the lesion and local nourishing blood vessels completely disappeared | |
| **Fu 2018^75^** | **2** | | China | | retrospective cohort | | 189 | | UACE + curettage under hysteroscopy vs curettage under ultrasonography vs laparoscopic cesarean scar resection | | 2 | | ND | |
| **Guo 2018^76^** | **2** | | China | | retrospective cohort | | 87 | | UAE + US guided D&C vs laparoscopic cesarean scar pregnancy debridement surgery | | 2 | | ND | |
| **Kim 2018^77^** | **1** | | South Korea | | retrospective cohort | | 58 | | Expectant vs MTX+/-KCL vs wedge resection laparotomy vs D&C vs hysteroscopy vs UAE vs hysterectomy | | 3 | | Sufficiently decreased hCG after first line treatment and therefore additional treatment not required | |
| **Lin 2018^78^** | **2** | | Taiwan | | retrospective cohort | | 109 | | TCR by hysteroscopy vs hysterotomy by either laparoscopy or mini-laparotomy and hysterectomy vs MTX | | 3 | | ND | |
| **Sun 2018^79^** | **1** | | China | | | retrospective cohort | | 395 | Suction evacuation following UACE (MTX + gelatin sponge) | 2 | | | | NR |
| **Wang Beej 2018^80^** | **1** | | | China | | retrospective cohort | | 107 | Ultrasound-guided suction curettage +/- MTX (IM or IV) | 4 | | | | No retained products of conception |
| **Zhang 2018^81^** | | **2** | | China | | retrospective cohort | | 76 | HIFU followed by ultrasound guided suction curettage | 3 | | | | NR |
| **Chen 2017^82^** | | **1** | | China | | retrospective cohort | | 76 | Transvaginal hysterotomy vs UAE combined with uterine curettage | 4 | | | | NR |
| **Hong 2017^83^** | | **2** | | China | | retrospective cohort | | 152 | Suction curettage under hysteroscopy prior to HIFU or UAE | 4 | | | | Control of bleeding and complete recovery with fertility preservation, without repeated embolisation, surgical intervention and without any severe complications |
| **Li 2017^84^** | | **2** | | China | | retrospective cohort | | 54 | Transvaginal clearance + balloon catheter vs hysteroscopic resection under US guidance +/- ballon catheter following IM MTX + oral mifepristone | 4 | | | | NR |
| **Liu 2017^85^** | | **1** | | China | | retrospective cohort | | 86 | UAE+MTX+D&C vs UAE+MTX vs D&C | 3 | | | | No need for additional treatment or a greater than 50% reduction in hCG |
| **Xiao 2017^86^** | | **2** | | China | | retrospective cohort | | 76 | HIFU vs UAE+MTX+D&C | 4 | | | | Complete recovery with no severe complications (such as severe bleeding, uterine rupture and gastrointestinal perforation), preservation of fertility and no need for repeat embolisation or hysterectomy |
| **Jurkovic 2016^87^** | | **1** | | UK | | retrospective cohort | | 232 | Ultrasound guidance using suction curettage +/- Shirodkar suture +/- pre local MTX +/- pre-UAE vs expectant management | 4 | | | | NR |
| **Li 2016^88^** | | **1** | | China | | retrospective cohort | | 52 | IM MTX + D&C vs UAE + D&C vs direct excision of CSP by laparotomy vs UAE + direct excision of CSP by laparotomy | 2 | | | | NR |
| **Liu 2016^89^** | | **1** | | China | | retrospective cohort | | 51 | Ultrasound guided D&C | 3 | | | | Disappearance of the mass, hCG dropped to zero, and no serious complications, such as uncontrollable bleeding, uterine rupture, and hysterectomy |
| **Yang 2016^90^** | | **1** | | China | | retrospective cohort | | 131 | UACE vs systemic methotrexate injection +/- D&C | 4 | | | | hCG decreased > 90% of the pretreatment value |
| **Zhu 2016^91^** | | **2** | | China | | retrospective cohort | | 122 | HIFU + suction curettage under hysteroscopic guidance vs UAE + suction curettage under hysteroscopic guidance | 3 | | | | NR |
| **Chen 2015^92^** | | **2** | | China | | retrospective cohort | | 128 | UAE vs transvaginal debridement and repair surgery +/- preoperative chemotherapy | 3 | | | | No ineffective treatment of UAE manifesting secondary vaginal haemorrhage |
| **Guo 2015^93^** | | **2** | | China | | retrospective cohort | | 78 | Laparotomy (hysterotomy + subtotal hysterectomy) vs curettage after UAE +/- MTX infusion vs D&C vs transvaginal sonographic guided local intragestational MTX injection vs systemic MTX injection | 4 | | | | ND |
| **Qi 2015^94^** | | **1** | | China | | retrospective cohort | | 50 | UAE receiving or not receiving local MTX infusion prior to ultrasound guided curettage | 4 | | | | ND |
| **Wu 2015^95^** | | **1** | | China | | retrospective cohort | | 232 | Transabdominal sonography-guided D&C | 4 | | | | Normal hCG without CSP mass, residual pregnancy tissue, or major complications requiring further treatments. Patients with a blood loss of >500 mL that was successfully stopped by Foley compression without residual tissue were also considered to have been treated successfully. |
| **Zhai 2015^96^** | | **2** | | China | | retrospective cohort | | 56 | Systemic MTX + mifepristone vs Local MTX vs lesion resection with hysteroscopy and laparoscopy+ repair of the caesarean scar vs removal of the embryo by cutting the vagina + repair operation of the caesarean scar | 3 | | | | NR |
| **Zhu 2015^97^** | | **2** | | China | | retrospective cohort | | 53 | HIFU followed by suction curettage under hysteroscopic guidance | 3 | | | | NR |
| **Cao 2014^98^** | | **2** | | China | | retrospective cohort | | 54 | UAE or hysteroscopy and suction curettage after bilateral UAE | 2 | | | | No hysterectomies |
| **Gao 2014^99^** | | **1** | | China | | retrospective cohort | | 119 | Systemic MTX and ultrasound guided D&C vs UAE combined with ultrasound guided D&C within 24 hours | 4 | | | | No need for further intervention after MTX and D&C or no need for further intervention (e.g. hysteroscopic resection of the lesion or injection of MTX) after UAE and D&C) |
| **He 2014^100^** | | **1** | | China | | retrospective cohort | | 58 | Intra-arterial MTX + UAE + laparoscopy + hysteroscopy + repair of defect vs intra-arterial MTX + UAE + US guided uterine curettage | 4 | | | | Complete or partial removal of the ectopic conceptus, cease of abnormal vaginal bleeding and preservation of the uterus |
| **Li 2014^101^** | | **1** | | China | | retrospective cohort | | 124 | Curettage by hysteroscopy under US vs MTX +curettage by hysteroscopy vs UAE +curettage by hysteroscopy | 3 | | | | No visible residue postop |
| **Shao 2014^102^** | | **1** | | China | | retrospective cohort | | 61 | MTX /leucovorin/UAE/UACE followed by uterine curettage under hysteroscopy guidance and ultrasound surveillance or hysteroscopy guidance and laparoscopy surveillance vs Lesion excision through abdomen and uterine repair | 4 | | | | NR |
| **Wang 2014^103^** | | **1** | | China | | retrospective cohort | | 71 | Oral mifepristone + hysteroscopic resection under US guidance + balloon catheter vs oral mifepristone + laparoscopic resection + balloon catheter | 3 | | | | ND |

CSEP, caesarean scar ectopic pregnancy; CSP, caesarean scar pregnancy; D&C, dilatation and curettage; hCG, human chorionic gonadotropin; HIFU, Focused Ultrasound Ablation Surgery; IM, intramuscular; GS, gestational sac; KCL, potassium chloride; LAOH, laparoscopy assisted by operative hysteroscopy; MTX, methotrexate; ND, not defined; NR, not reported; NS, not specified; RCT, randomised controlled trial; TAH, total abdominal hysterectomy; TCR, transcervical resection; UACE, uterine artery chemoembolisation; UAE, uterine artery embolisation, US, ultrasound; USG-LL, Ultrasound-Guided Local Lauromacrogol Injection.

**Table S3 Outcome reporting in CSEP trials: Randomised controlled trials (n=5)**

| **Author, year** | **Journal**  **(1=specialist,**  **2=general)** | **Country** | **Sample size** | **Aim** | **Gestation at treatment** | **Method of diagnosis** | **Intervention(s)** | **Primary outcome** | **Outcome quality score** | **Definition of successful treatment** |
| --- | --- | --- | --- | --- | --- | --- | --- | --- | --- | --- |
| **Di Spiezio Sardo 2023^104^** | **1** | Italy | 54 | To compare the success rate of hysteroscopic resection vs ultrasound-guided D&E for the treatment of CSP | 1st trimester | TVS | IM MTX + hysteroscopic resection or ultrasound-guided D&E | Success rate | 5 | No further treatment required until the complete resolution of CSP, based on decline of hCG and the absence of residual gestational material in the endometrial cavity |
| **Yu 2021^105^** | **1** | China | 61 | To evaluate the optimal time interval between UAE and D&C regimen, that is, whether the procedure is completed on the same day or additional hospitalization, and to identify the most effective treatment | 1^st^ trimester | Ultrasound, MRI | UAE within 12h vs 12-72h followed by D&C | NS | 4 | ND |
| **Li Gong 2016^106^** | **1** | China | 144 | To compare the complication rates after UACE followed by D&C guided by different types of monitoring for treatment of CSP | NS | Ultrasound | UACE + D&C with hysteroscopy monitoring or ultrasonography monitoring or no monitoring | Number of participants with short-term complications at 2 months after D&C | 6 | NR |
| **Peng 2015^107^** | **2** | China | 104 | To investigate the MTX treatment in CSP patients prospectively; to compare the efficacy between local injection and systemic administration of MTX, and to analyse the factors in favour of patient prognosis, providing reference for screening candidates for MTX therapy | 1st trimester | Ultrasound | Local vs systemic MTX | NS | 4 | Regression of ultrasonographic findings and normalisation of hCG within 60 days |
| **Qian 2015^108^** | **1** | China | 66 | To compare the clinical efficacy and safety of two managements, and to explore the necessity of operative hysteroscopy for the treatment of the gestational sac type of CSP following preventive UAE | 1st trimester | Ultrasound | UAE + D&C vs UAE + operative hysteroscopy + curettage | NS | 4 | ND |

CSP, caesarean scar pregnancy; D&C, dilatation and curettage; D&E, dilation and evacuation; hCG, human chorionic gonadotropin; IM, intramuscular; MRI, Magnetic resonance imaging; MTX, methotrexate; ND, not defined; NR, not reported; NS, not specified; TVS, transvaginal ultrasound; UACE, uterine artery chemoembolisation; UAE, uterine artery embolisation.

**Table S4 Outcome reporting in CSEP trials: Risk of bias assessment using The Evidence Project risk of bias tool**

| **Author, year** | **Cohort** | **Control or comparison group?** | **Pre- and postintervention data provided?** | **Random assignment of participants to intervention?** | **Random selection of participants for assessment?** | **Follow up rate 80% or more?** | **Comparison groups equivalent on demographics** | **Comparison groups equivalent at baseline on outcome measures** |
| --- | --- | --- | --- | --- | --- | --- | --- | --- |
|  | **Study Design** | | | **Participant Representativeness** | | | **Equivalence of comparison groups** | |
| **Ban 2023** | Yes | Yes | No | N/A | No | Yes | No | Yes |
| **Cagli 2023** | Yes | No | Yes | N/A | No | Yes | N/A | N/A |
| **Cao 2014** | Yes | No | Yes | N/A | No | Yes | N/A | N/A |
| **Cao 2021** | Yes | Yes | Yes | N/A | No | Yes | No | Yes |
| **Cao 2022** | Yes | Yes | Yes | N/A | No | Yes | Yes | Yes |
| **Chen 2015** | Yes | Yes | Yes | N/A | No | Yes | Yes | Yes |
| **Chen 2017** | Yes | Yes | Yes | N/A | No | Yes | Yes | Yes |
| **Chen 2021** | Yes | Yes | No | N/A | No | Yes | Yes | No |
| **Chen 2022** | Yes | No | Yes | N/A | No | Yes | N/A | N/A |
| **Cheng 2020** | Yes | Yes | Yes | N/A | No | Yes | Yes | No |
| **De Braud 2021** | Yes | No | No | N/A | No | Yes | N/A | N/A |
| **Di Spiezio Sardo 2023** | Yes | Yes | Yes | Yes | No | Yes | Yes | Yes |
| **Fang 2020** | Yes | Yes | Yes | N/A | No | Yes | No | No |
| **Fei 2019** | Yes | Yes | No | N/A | No | Yes | Yes | Yes |
| **Fu 2018** | Yes | Yes | No | N/A | No | Yes | NR | NR |
| **Fu 2022** | Yes | Yes | Yes | N/A | No | NR | Yes | Yes |
| **Fu 2023** | Yes | No | No | N/A | No | Yes | N/A | N/A |
| **Gao 2014** | Yes | Yes | Yes | N/A | No | NR | Yes | Yes |
| **Gu 2022** | Yes | No | Yes | N/A | No | Yes | N/A | N/A |
| **Gui 2017** | Yes | Yes | No | N/A | No | NR | No | N/A |
| **Guo 2015** | Yes | Yes | Yes | N/A | No | No | NR | Yes |
| **Guo 2018** | Yes | Yes | No | N/A | No | Yes | Yes | Yes |
| **Harb 2018** | Yes | Yes | No | No | No | Yes | NR | NR |
| **He 2014** | Yes | Yes | Yes | N/A | No | Yes | Yes | Yes |
| **Hong 2017** | Yes | Yes | Yes | N/A | No | Yes | Yes | Yes |
| **Hong 2022** | Yes | Yes | No | N/A | No | Yes | No | NR |
| **Huang Li 2020** | Yes | Yes | No | N/A | No | Yes | Yes | NR |
| **Huang Zhao 2020** | Yes | Yes | No | N/A | No | Yes | Yes | NR |
| **Huo 2023** | Yes | Yes | Yes | N/A | No | NR | No | No |
| **Jurkovic 2016** | Yes | Yes | No | N/A | No | No | Yes | Yes |
| **Kaelin Agten 2023** | Yes | Yes | No | N/A | No | Yes | NR | NR |
| **Kim 2018** | Yes | Yes | No | N/A | No | Yes | NR | NR |
| **Lan 2021** | Yes | No | No | N/A | No | Yes | N/A | N/A |
| **Le 2019** | Yes | Yes | Yes | N/A | No | Yes | Yes | No |
| **Levin 2021** | Yes | Yes | Yes | N/A | No | Yes | Yes | Yes |
| **Li 2014** | NR | Yes | Yes | N/A | No | Yes | Yes | No |
| **Li 2016** | Yes | Yes | Yes | N/A | No | Yes | No | Yes |
| **Li 2017** | Yes | Yes | Yes | N/A | No | Yes | Yes | Yes |
| **Li Gong 2016** | No | Yes | Yes | Yes | No | Yes | Yes | Yes |
| **Lin 2018** | Yes | Yes | No | N/A | No | Yes | No | NR |
| **Lin 2021** | Yes | Yes | No | N/A | No | Yes | Yes | Yes |
| **Liu 2016** | Yes | No | Yes | N/A | No | Yes | N/A | N/A |
| **Liu 2017** | Yes | Yes | Yes | N/A | No | Yes | Yes | Yes |
| **Liu 2020** | Yes | Yes | Yes | N/A | No | Yes | Yes | Yes |
| **Liu 2021** | Yes | Yes | No | N/A | No | Yes | Yes | Yes |
| **Liu 2022** | Yes | No | Yes | N/A | No | Yes | N/A | N/A |
| **Liu Xu 2022** | Yes | Yes | Yes | N/A | No | Yes | Yes | Yes |
| **Lu 2020** | Yes | Yes | Yes | N/A | No | Yes | Yes | Yes |
| **Lu 2023** | Yes | Yes | Yes | N/A | No | Yes | Yes | Yes |
| **Meyer 2023** | Yes | Yes | Yes | N/A | No | Yes | Yes | Yes |
| **Mitsui 2021** | Yes | Yes | No | N/A | No | Yes | NR | NR |
| **Mu 2023** | Yes | No | Yes | N/A | No | Yes | N/A | N/A |
| **Ou 2020** | Yes | Yes | Yes | No | No | Yes | Yes | Yes |
| **Peng 2015** | Yes | Yes | Yes | Yes | No | Yes | Yes | Yes |
| **Peng 2022** | Yes | No | Yes | N/A | No | Yes | N/A | N/A |
| **Qi 2015** | Yes | Yes | No | N/A | No | Yes | Yes | Yes |
| **Qian 2015** | Yes | Yes | Yes | Yes | No | Yes | Yes | Yes |
| **Qiu 2019** | Yes | Yes | Yes | N/A | No | NR | Yes | Yes |
| **Qiuyang 2019** | Yes | No | Yes | N/A | No | NR | N/A | N/A |
| **Qu 2022** | Yes | Yes | Yes | N/A | No | NR | No | Yes |
| **Shao 2014** | Yes | Yes | Yes | N/A | No | NR | NR | NR |
| **Shao 2022** | Yes | Yes | Yes | N/A | No | NR | Yes | Yes |
| **Shen 2021** | Yes | Yes | Yes | N/A | No | NR | Yes | No |
| **Shen 2023** | Yes | Yes | Yes | N/A | No | Yes | Yes | Yes |
| **Shiber 2023** | Yes | Yes | No | N/A | No | No | NR | No |
| **Spong 2023** | Yes | Yes | No | N/A | No | Yes | Yes | NR |
| **Sun 2018** | Yes | No | Yes | N/A | No | Yes | N/A | N/A |
| **Sun 2021** | NR | Yes | No | N/A | No | No | Yes | N/A |
| **Sun 2023** | Yes | Yes | Yes | No | No | Yes | Yes | Yes |
| **Tan 2022** | Yes | Yes | Yes | No | No | Yes | Yes | No |
| **Tan Sun 2020** | Yes | Yes | No | N/A | No | NR | No | NR |
| **Tang 2021** | Yes | Yes | Yes | N/A | No | Yes | No | Yes |
| **Tarafdari 2024** | Yes | Yes | Yes | No | No | Yes | NR | NR |
| **Timor-Tritsch 2015** | NR | Yes | No | N/A | No | Yes | NR | NR |
| **Van 2023** | Yes | No | Yes | N/A | No | Yes | N/A | N/A |
| **Verberkt 2023** | Yes | Yes | Yes | N/A | No | Yes | No | No |
| **Wang 2014** | Yes | Yes | Yes | N/A | No | NR | Yes | Yes |
| **Wang 2015** | No | Yes | No | N/A | No | NR | No | No |
| **Wang 2018** | Yes | Yes | Yes | N/A | No | NR | NR | Yes |
| **Wang 2022** | Yes | Yes | No | N/A | No | Yes | Yes | No |
| **Wang 2023** | Yes | Yes | Yes | N/A | No | NR | Yes | Yes |
| **Wang Beej 2018** | Yes | Yes | No | N/A | No | NR | No | No |
| **Wu 2014** | Yes | Yes | Yes | No | No | Yes | Yes | Yes |
| **Wu 2015** | Yes | No | No | N/A | No | NR | N/A | N/A |
| **Wu 2020** | Yes | Yes | Yes | N/A | No | Yes | Yes | Yes |
| **Wu 2021** | NR | Yes | Yes | N/A | No | NR | Yes | Yes |
| **Wu 2023** | Yes | Yes | Yes | N/A | No | NR | Yes | Yes |
| **Wu Guo 2023** | Yes | Yes | Yes | Yes | No | Yes | Yes | Yes |
| **Xiao 2017** | Yes | Yes | Yes | N/A | No | Yes | Yes | Yes |
| **Xiao 2019** | Yes | Yes | Yes | N/A | No | NR | Yes | No |
| **Xiong 2021** | Yes | Yes | Yes | N/A | No | NR | No | No |
| **Xu 2021** | Yes | Yes | Yes | N/A | No | Yes | No | Yes |
| **Xu 2022** | Yes | Yes | No | N/A | No | No | Yes | N/A |
| **Yang 2016** | Yes | Yes | Yes | N/A | No | NR | NR | NR |
| **Yang 2023** | Yes | Yes | No | N/A | No | NR | No | NR |
| **Yang Cao 2023** | Yes | Yes | No | N/A | No | NR | Yes | NR |
| **Yin 2020** | Yes | Yes | No | N/A | No | NR | No | NR |
| **Yu 2021** | Yes | Yes | Yes | Yes | No | Yes | Yes | Yes |
| **Yuan 2021** | Yes | No | Yes | N/A | No | NR | N/A | N/A |
| **Zeng 2023** | NR | Yes | Yes | N/A | No | NR | Yes | No |
| **Zhai 2015** | NR | Yes | Yes | N/A | No | NR | Yes | No |
| **Zhang 2017** | Yes | Yes | No | N/A | No | NR | Yes | Yes |
| **Zhang 2018** | Yes | No | Yes | N/A | No | NR | N/A | N/A |
| **Zhang 2020** | Yes | Yes | Yes | N/A | No | NR | No | Yes |
| **Zhou 2022** | Yes | Yes | Yes | N/A | No | Yes | Yes | Yes |
| **Zhu 2015** | Yes | No | Yes | N/A | No | NR | N/A | N/A |
| **Zhu 2016** | Yes | Yes | Yes | N/A | No | NR | Yes | Yes |
| **Zhu 2022** | Yes | Yes | Yes | N/A | No | Yes | Yes | Yes |

N/A, not applicable; NR, not recorded

1. Tarafdari A, Hadizadeh A, Irandoost E, Borna S, Ghamari A, Ghotbizadeh Vahdani F. Cesarean Scar Pregnancy: Results of Treatment Using a Double-Balloon Cervical Ripening Catheter. J Obstet Gynaecol India. 2024;74(1):71-9.

2. Fu L, Yuan H, Cao H, Zhou Q, Tan X, Guo J. Clinical value of ultrasonic indicators in predicting the outcome of caesarean scar pregnancy after pregnancy termination. BMC Pregnancy Childbirth. 2023;23(1):863.

3. Cao L, Qian Z, Huang L. Comparison of D&C and hysteroscopy after UAE in the treatment of cesarean scar pregnancy: A case-control study. Medicine (Baltimore). 2022;101(3):e28607.

4. Liu T, Xu X. Comparison of efficacies between ultrasound-guided curettage combined with hysteroscopic electro-resection after injection of pituitrin and hysteroscopic electro-resection after methotrexate chemotherapy in the treatment of cesarean scar pregnancy. J Obstet Gynaecol. 2022;42(7):3041-7.

5. Wang J, Wang D, Zhang X, Liu Y, Yang Q, Zhang N. The effect of prophylactic uterine artery embolization on reproductive outcomes in patients with cesarean scar pregnancy: a propensity score-matched study. Arch Gynecol Obstet. 2022;305(3):651-9.

6. Zhu W, Zhang X, Liu C, Liu Y, Xu W. Uterine Artery Embolization on Serum β-HCG Levels, Fertility Function and Clinical Efficacy in Patients With Cesarean Uterine Scar Pregnancy. Front Surg. 2022;9:838879.

7. Liu Z, Shi Z, Wei Y, Dai Q, Liu X. Lacunar-like changes of the chorion: can it be a first-trimester ultrasound sign in predicting worse clinical outcome in cesarean scar pregnancy termination? J Matern Fetal Neonatal Med. 2021;34(14):2355-62.

8. Liu CN, Tang L, Sun Y, Liu YH, Yu HJ. Clinical outcome of high-intensity focused ultrasound as the preoperative management of cesarean scar pregnancy. Taiwan J Obstet Gynecol. 2020;59(3):387-91.

9. Lu YM, Guo YR, Zhou MY, Wang Y. Indwelling Intrauterine Foley Balloon Catheter for Intraoperative and Postoperative Bleeding in Cesarean Scar Pregnancy. J Minim Invasive Gynecol. 2020;27(1):94-9.

10. Xiao Z, Cheng D, Chen J, Yang J, Xu W, Xie Q. The effects of methotrexate and uterine arterial embolization in patients with cesarean scar pregnancy: A retrospective case-control study. Medicine (Baltimore). 2019;98(11):e14913.

11. Wang A-L, Chen Y-X, Cao L-X. Clinical observation of hysteroscopic surgery combined with ectopic pregnancy ii decoction and methotrexate in the treatment of cesarean scar pregnancy. Journal of Hainan Medical University. 2018;24(10):34-8.

12. Gui T, Peng P, Liu X, Jin L, Chen W. Clinical and ultrasound parameters in prediction of excessive hemorrhage during management of cesarean scar pregnancy. Ther Clin Risk Manag. 2017;13:807-12.

13. Zhang H, Huang J, Wu X, Fan H, Li H, Gao T. Clinical classification and treatment of cesarean scar pregnancy. J Obstet Gynaecol Res. 2017;43(4):653-61.

14. Wang Q, Ma H, Peng H, He L, Bian C, Zhao X. Risk factors for intra-operative haemorrhage and bleeding risk scoring system for caesarean scar pregnancy: a case-control study. Eur J Obstet Gynecol Reprod Biol. 2015;195:141-5.

15. Sun J, Peng C, Liu X, Lv Y, Shen H, Xu Z, et al. Effects of lauromacrogol injection under contrast-enhanced ultrasound guidance on cesarean scar pregnancy: a prospective cohort study. Quant Imaging Med Surg. 2023;13(3):1849-59.

16. Wu J, Guo R, Li L, Chu D, Wang X. Effectiveness and safety of prophylactic abdominal aortic balloon occlusion for patients with type III caesarean scar pregnancy: a prospective cohort study. BMC Pregnancy Childbirth. 2023;23(1):754.

17. Tan KL, Chen YM, Zeng W, Meng Y, Jiang L. Local Methotrexate Injection Followed by Dilation and Curettage for Cesarean Scar Pregnancy: A Prospective Non-randomized Study. Front Med (Lausanne). 2021;8:800610.

18. Sun X, Liu Y, Tang Y, Yu H, Zhao M, Chen Q. Subsequent Fertility in Women Treated for Caesarean Scar Pregnancy With Hysteroscopy: A 5-Year Follow-Up Descriptive Study in a Tertiary Hospital. Front Endocrinol (Lausanne). 2021;12:659647.

19. Ou J, Peng P, Li C, Teng L, Liu X. Assessment of the necessity of uterine artery embolization during suction and curettage for caesarean scar pregnancy: a prospective cohort study. BMC Pregnancy Childbirth. 2020;20(1):378.

20. Harb HM, Knight M, Bottomley C, Overton C, Tobias A, Gallos ID, et al. Caesarean scar pregnancy in the UK: a national cohort study. Bjog. 2018;125(13):1663-70.

21. Timor-Tritsch IE, Khatib N, Monteagudo A, Ramos J, Berg R, Kovács S. Cesarean scar pregnancies: experience of 60 cases. J Ultrasound Med. 2015;34(4):601-10.

22. Wu X, Xue X, Wu X, Lin R, Yuan Y, Wang Q, et al. Combined laparoscopy and hysteroscopy vs. uterine curettage in the uterine artery embolization-based management of cesarean scar pregnancy: a cohort study. Int J Clin Exp Med. 2014;7(9):2793-803.

23. Ban Y, Shen J, Wang X, Zhang T, Lu X, Qu W, et al. Cesarean Scar Ectopic Pregnancy Clinical Classification System With Recommended Surgical Strategy. Obstet Gynecol. 2023;141(5):927-36.

24. Cagli F, Dolanbay M, Gülseren V, Kütük S, Aygen EM. Is local methotrexate therapy effective in the treatment of cesarean scar pregnancy? A retrospective cohort study. J Obstet Gynaecol Res. 2023;49(1):122-7.

25. Huo S, Shen L, Ju Y, Liu K, Liu W. Treatments for cesarean scar pregnancy: 11-year experience at a medical center. J Matern Fetal Neonatal Med. 2023;36(1):2162818.

26. Kaelin Agten A, Jurkovic D, Timor-Tritsch I, Jones N, Johnson S, Monteagudo A, et al. First-trimester cesarean scar pregnancy: a comparative analysis of treatment options from the international registry. Am J Obstet Gynecol. 2023.

27. Lu L, Shao Y, Qu Z, Huang G, Lang S, Yang C, et al. Outcomes of prophylactic lauromacrogol injection versus non-injection in patients with endogenous cesarean scar pregnancy treated by hysteroscopic surgery: a retrospective cohort study. BMC Pregnancy Childbirth. 2023;23(1):771.

28. Meyer R, Friedrich L, Plaschkes R, Toussia-Cohen S, Levin G, Weissbach T, et al. Clinical implications of a cesarean scar pregnancy sonographic evaluation and reporting system. Eur J Obstet Gynecol Reprod Biol. 2023;291:247-51.

29. Mu L, Weng H, Dong Y. Analysis of risk factors for patients with cesarean scar pregnancy treated with methotrexate combined with suction curettage. Arch Gynecol Obstet. 2023;308(3):913-8.

30. Shen M, Li L, Zhu L, Liu J, Lin T, Liu X. Predictive value of crossover sign for outcome of ultrasound-guided vacuum aspiration in women with Cesarean scar pregnancy. Ultrasound Obstet Gynecol. 2024;63(4):544-50.

31. Shiber Y, Maymon R, Gal-Kochav M, Kugler N, Pekar-Zlotin M, Smorgick N, et al. Caesarean scar pregnancy: is there a light in the end of the tunnel? Arch Gynecol Obstet. 2023;307(4):1057-64.

32. Spong CY, Yule CS, Fleming ET, Lafferty AK, McIntire DD, Twickler DM. The Cesarean Scar of Pregnancy: Ultrasound Findings and Expectant Management Outcomes. Am J Perinatol. 2024;41(S 01):e1445-e50.

33. Van X, Bui T, Dinh HT, Van T, Tran A. The Effectiveness of Combined Local and Systemic Methotrexate Treatment in Cesarean Scar Pregnancy Weeks 8 to 14. Gynecol Minim Invasive Ther. 2023;12(3):170-4.

34. Verberkt C, Lemmers M, de Leeuw RA, van Mello NM, Groenman FA, Hehenkamp WJK, et al. Effectiveness, complications, and reproductive outcomes after cesarean scar pregnancy management: a retrospective cohort study. AJOG Glob Rep. 2023;3(1):100143.

35. Wang X, Yang B, Chen W, Chen J. Clinical efficacy and re-pregnancy outcomes of patients with previous cesarean scar pregnancy treated with either high-intensity focused ultrasound or uterine artery embolization before ultrasound-guided dilatation and curettage: a retrospective cohort study. BMC Pregnancy Childbirth. 2023;23(1):85.

36. Wu T, Wang Q, Liu W, Zhang J, Wang W, Wang J, et al. Clinical efficacy and risk factors for suction curettage and hysteroscopy in patients with type I and II cesarean scar pregnancy. Int J Gynaecol Obstet. 2024;164(1):270-6.

37. Yang X, Zheng W, Wei X, Ma J, Yan J, Poon LC, et al. Management of cesarean scar pregnancy: Importance of gestational age at diagnosis and disease type-A single center's 5 years of experience involving 223 cases. Front Surg. 2023;10:1055245.

38. Yang M, Cao L, Yan J, Tang Y, Cao N, Huang L. Risk factors associated with the failure of initial treatment for cesarean scar pregnancy. Int J Gynaecol Obstet. 2023;162(3):937-44.

39. Zeng S, Wang Y, Ye P, Xu L, Han W, Li F, et al. Comparing the clinical efficacy of three surgical methods for cesarean scar pregnancy. BMC Womens Health. 2023;23(1):271.

40. Chen YT, Chen YC, Chen M, Chang YJ, Yang SH, Tsai HD, et al. Reproductive outcomes of cesarean scar pregnancies treated with uterine artery embolization combined with curettage. Taiwan J Obstet Gynecol. 2022;61(4):601-5.

41. Fu P, Zhou T, Cui P, Wang W, Wang S, Liu R. Selection of Laparoscopy or Laparotomy for Treating Cesarean Scar Pregnancy: A Retrospective Study. Int J Gen Med. 2022;15:7229-40.

42. Gu Z, Jia P, Gao Z, Gu W, Zhao H, Zhao S. Uterine artery embolization combined with ultrasound-guided dilation and curettage for the treatment of cesarean scar pregnancy: Efficacy and 5-8-year follow-up study. J Interv Med. 2022;5(3):148-52.

43. Hong T, Chai Z, Liu M, Zheng L, Qi F. The Efficacy and Health Economics of Different Treatments for Type 1 Cesarean Scar Pregnancy. Front Pharmacol. 2022;13:822319.

44. Liu Y, Yin Q, Xu F, Luo S. Clinical efficacy and safety of high-intensity focused ultrasound (HIFU) ablation in treatment of cesarean scar pregnancy (CSP) I and II. BMC Pregnancy Childbirth. 2022;22(1):607.

45. Peng Y, Dai Y, Yu G, Jin P. Analysis of the type of cesarean scar pregnancy impacted on the effectiveness and safety of high intensity focused ultrasound combined with ultrasound-guided suction curettage treatment. Int J Hyperthermia. 2022;39(1):1449-57.

46. Qu W, Li H, Zhang T, Zhang Y, Ban Y, Li N, et al. Comparison of different treatment strategies in the management of endogenic caesarean scar pregnancy: a multicentre retrospective study. BMC Pregnancy Childbirth. 2022;22(1):404.

47. Shao M, Tang F, Ji L, Hu M, Zhang K, Pan J. The management of caesarian scar pregnancy with or without a combination of methods prior to hysteroscopy: Ovarian reserve trends and patient outcomes. J Gynecol Obstet Hum Reprod. 2022;51(8):102417.

48. Xu Z, Sheng C, Yang Q, Wang J. Analysis of pregnancy outcomes following surgical treatment of cesarean scar pregnancy. BMC Pregnancy Childbirth. 2022;22(1):644.

49. Zhou W, Feng X, Yu J, Chai Z, Zheng L, Qi F. The efficacy of different treatments for type 2 cesarean scar pregnancy. Fertil Steril. 2022;118(2):407-13.

50. Cao S, Qiu G, Zhang P, Wang X, Wu Q. A Comparison of Transvaginal Removal and Repair of Uterine Defect for Type II Cesarean Scar Pregnancy and Uterine Artery Embolization Combined With Curettage. Front Med (Lausanne). 2021;8:654956.

51. Chen R, An J, Guo Q, Lin Q, Yang L, Wang Y. Temporary Ligation of the Bilateral Uterine Arteries During Laparoscopy Combined with Hysteroscopy in the Treatment of Caesarean Scar Pregnancy: Experience at a Chinese Teaching Hospital. Int J Gen Med. 2021;14:2087-94.

52. De Braud LV, Knez J, Mavrelos D, Thanatsis N, Jauniaux E, Jurkovic D. Risk prediction of major haemorrhage with surgical treatment of live cesarean scar pregnancies. Eur J Obstet Gynecol Reprod Biol. 2021;264:224-31.

53. Lan CF, Lin BH, Nie CL, Guo XL, Zhou DH, Lin YJ. Investigation and Analysis of Influencing Factors of Moderate to Severe Pain After Uterine Artery Embolization in Patients with Cesarean Scar Pregnancy. Int J Gen Med. 2021;14:9023-9.

54. Levin G, Shai D, Dior UP, Gilad R, Shushan A, Benshushan A, et al. Single- versus multiple-dose methotrexate in cesarean scar pregnancies management: treatment and reproductive outcomes. Arch Gynecol Obstet. 2021;303(5):1255-61.

55. Lin Y, Xiong C, Dong C, Yu J. Approaches in the Treatment of Cesarean Scar Pregnancy and Risk Factors for Intraoperative Hemorrhage: A Retrospective Study. Front Med (Lausanne). 2021;8:682368.

56. Mitsui T, Mishima S, Tani K, Maki J, Eto E, Hayata K, et al. Clinical Course of 60 Cesarean Scar Pregnancies. Acta Med Okayama. 2021;75(4):439-45.

57. Shen F, Lv H, Wang L, Zhao R, Tong M, Lee AC, et al. A Comparison of Treatment Options for Type 1 and Type 2 Caesarean Scar Pregnancy: A Retrospective Case Series Study. Front Med (Lausanne). 2021;8:671035.

58. Tang Q, Qin Y, Zhou Q, Tang J, Zhou Q, Qiao J, et al. Hysteroscopic treatment and reproductive outcomes in cesarean scar pregnancy: experience at a single institution. Fertil Steril. 2021;116(6):1559-66.

59. Wu Y, Sun LF, Si YN, Luan XL, Gao YM. Clinical efficacy analysis of different therapeutic methods in patients with cesarean scar pregnancy. Taiwan J Obstet Gynecol. 2021;60(3):498-502.

60. Xiong J, Fu F. Study on influencing factors and related clinical issues in cesarean scar pregnancy. 2021.

61. Xu X, Li D, Yang L, Jing X, Kong X, Chen D, et al. Surgical outcomes of cesarean scar pregnancy: an 8-year experience at a single institution. Arch Gynecol Obstet. 2021;303(5):1223-33.

62. Yuan Y, Pu D, Zhan P, Zheng Y, Ren Q, Teichmann AT. Focused Ultrasound Ablation Surgery combined with ultrasound-guided suction curettage in the treatment and management of Cesarean Scar Pregnancy. Eur J Obstet Gynecol Reprod Biol. 2021;258:168-73.

63. Cheng Q, Tian Q, Chang K-K, Yi X-F. Comparison of the efficacy and safety of different surgical strategies for patients with type II cesarean scar pregnancy. Reproductive and Developmental Medicine. 2020;4(02):89-96.

64. Fang S, Zhang P, Zhu Y, Wang F, He L. A Retrospective Analysis of the Treatment of Cesarean Scar Pregnancy by High-Intensity Focused Ultrasound, Uterine Artery Embolization and Surgery. Front Surg. 2020;7:23.

65. JR Huang J, X Li XL. Is preprocessing helpful for suction and curettage in treating cesarean scar pregnancy? 2020.

66. Huang L, Zhao L, Shi H. Clinical Efficacy of Combined Hysteroscopic and Laparoscopic Surgery and Reversible Ligation of the Uterine Artery for Excision and Repair of Uterine Scar in Patients with Type II and III Cesarean Scar Pregnancy. Med Sci Monit. 2020;26:e924076.

67. Tan TT, Sun QL, Luo L, Chen Z, Xiong X, Xiang JH, et al. Validation of a 10-Point Scoring System for Treatment of Cesarean Scar Pregnancy. Ther Clin Risk Manag. 2020;16:429-36.

68. Wu Q, Liu X, Zhu L, Zhu Y, Mei T, Cao S, et al. Clinical Assessment of Ultrasound-Guided Local Lauromacrogol Injection Combined With Curettage and Hysteroscopy for Cesarean Scar Pregnancy. Front Pharmacol. 2020;11:601977.

69. Yin X, Huang S. Clinical characteristics and treatment of different types of cesarean scar pregnancy. Ginekol Pol. 2020;91(7):406-11.

70. Zhang X, Pang Y, Ma Y, Liu X, Cheng L, Ban Y, et al. A comparison between laparoscopy and hysteroscopy approach in treatment of cesarean scar pregnancy. Medicine (Baltimore). 2020;99(43):e22845.

71. Fei H, Jiang X, Li T, Pan Y, Guo H, Xu X, et al. Comparison Of Three Different Treatment Methods For Cesarean Scar Pregnancy. Ther Clin Risk Manag. 2019;15:1377-81.

72. Le A, Li M, Xu Y, Wang Z, Dai XY, Xiao TH, et al. Different Surgical Approaches to 313 Cesarean Scar Pregnancies. J Minim Invasive Gynecol. 2019;26(1):148-52.

73. Qiu J, Fu Y, Xu J, Huang X, Yao G, Lu W. Analysis on clinical effects of dilation and curettage guided by ultrasonography versus hysteroscopy after uterine artery embolization in the treatment of cesarean scar pregnancy. Ther Clin Risk Manag. 2019;15:83-9.

74. Li Q, Xu H, Wang Y, Liu Q, He P, Wang L. Ultrasound-guided local methotrexate treatment for cesarean scar pregnancy in the first trimester: 12 years of single-center experience in China. Eur J Obstet Gynecol Reprod Biol. 2019;243:162-7.

75. Fu LP. Therapeutic approach for the cesarean scar pregnancy. Medicine (Baltimore). 2018;97(18):e0476.

76. Guo J, Yu J, Zhang Q, Song X. Clinical Efficacy and Safety of Uterine Artery Embolization (UAE) versus Laparoscopic Cesarean Scar Pregnancy Debridement Surgery (LCSPDS) in Treatment of Cesarean Scar Pregnancy. Med Sci Monit. 2018;24:4659-66.

77. Kim SY, Yoon SR, Kim MJ, Chung JH, Kim MY, Lee SW. Cesarean scar pregnancy; Diagnosis and management between 2003 and 2015 in a single center. Taiwan J Obstet Gynecol. 2018;57(5):688-91.

78. Lin SY, Hsieh CJ, Tu YA, Li YP, Lee CN, Hsu WW, et al. New ultrasound grading system for cesarean scar pregnancy and its implications for management strategies: An observational cohort study. PLoS One. 2018;13(8):e0202020.

79. Sun QL, Wu XH, Luo L, Ying DM, Yang Y, Chen ZQ. Characteristics of women with mixed mass formation after evacuation following uterine artery chemoembolization for cesarean scar pregnancy. Arch Gynecol Obstet. 2018;297(4):1059-66.

80. Wang S, Beejadhursing R, Ma X, Li Y. Management of Caesarean scar pregnancy with or without methotrexate before curettage: human chorionic gonadotropin trends and patient outcomes. BMC Pregnancy Childbirth. 2018;18(1):289.

81. Zhang Y, Zhang C, He J, Bai J, Zhang L. The impact of gestational sac size on the effectiveness and safety of high intensity focused ultrasound combined with ultrasound-guided suction curettage treatment for caesarean scar pregnancy. Int J Hyperthermia. 2018;35(1):291-7.

82. Chen H, Zhou J, Wang H, Tan W, Yao M, Wang X. The Treatment of Cesarean Scar Pregnancy with Uterine Artery Embolization and Curettage as Compared to Transvaginal Hysterotomy. Eur J Obstet Gynecol Reprod Biol. 2017;214:44-9.

83. Hong Y, Guo Q, Pu Y, Lu D, Hu M. Outcome of high-intensity focused ultrasound and uterine artery embolization in the treatment and management of cesarean scar pregnancy: A retrospective study. Medicine (Baltimore). 2017;96(30):e7687.

84. Li YY, Yin ZY, Li S, Xu H, Zhang XP, Cheng H, et al. Comparison of transvaginal surgery and methotrexate/mifepristone-combined transcervical resection in the treatment of cesarean scar pregnancy. Eur Rev Med Pharmacol Sci. 2017;21(12):2957-63.

85. Liu G, Wu J, Cao J, Xue Y, Dai C, Xu J, et al. Comparison of three treatment strategies for cesarean scar pregnancy. Arch Gynecol Obstet. 2017;296(2):383-9.

86. Xiao J, Shi Z, Zhou J, Ye J, Zhu J, Zhou X, et al. Cesarean Scar Pregnancy: Comparing the Efficacy and Tolerability of Treatment with High-Intensity Focused Ultrasound and Uterine Artery Embolization. Ultrasound Med Biol. 2017;43(3):640-7.

87. Jurkovic D, Knez J, Appiah A, Farahani L, Mavrelos D, Ross JA. Surgical treatment of Cesarean scar ectopic pregnancy: efficacy and safety of ultrasound-guided suction curettage. Ultrasound Obstet Gynecol. 2016;47(4):511-7.

88. Li Y, Wang W, Yang T, Wei X, Yang X. Incorporating uterine artery embolization in the treatment of cesarean scar pregnancy following diagnostic ultrasonography. Int J Gynaecol Obstet. 2016;134(2):202-7.

89. Liu S, Sun J, Cai B, Xi X, Yang L, Sun Y. Management of Cesarean Scar Pregnancy Using Ultrasound-Guided Dilation and Curettage. J Minim Invasive Gynecol. 2016;23(5):707-11.

90. Yang H, Li S, Ma Z, Jia Y. Therapeutic effects of uterine artery embolisation (UAE) and methotrexate (MTX) conservative therapy used in treatment of cesarean scar pregnancy. Arch Gynecol Obstet. 2016;293(4):819-23.

91. Zhu X, Deng X, Xiao S, Wan Y, Xue M. A comparison of high-intensity focused ultrasound and uterine artery embolisation for the management of caesarean scar pregnancy. Int J Hyperthermia. 2016;32(2):144-50.

92. Chen YQ, Liu HS, Li WX, Deng C, Hu XW, Kuang PJ. Efficacy of transvaginal debridement and repair surgery for cesarean scar pregnancy: a cohort study compared with uterine artery embolism. Int J Clin Exp Med. 2015;8(11):21187-93.

93. Guo MH, Wang MF, Liu MM, Qi F, Qu F, Zhou JH. Management of Cesarean Scar Pregnancy: A Case Series. Chin Med Sci J. 2015;30(4):226-30.

94. Qi F, Zhou W, Wang MF, Chai ZY, Zheng LZ. Uterine artery embolization with and without local methotrexate infusion for the treatment of cesarean scar pregnancy. Taiwan J Obstet Gynecol. 2015;54(4):376-80.

95. Wu XQ, Zhang HW, Fang XL, Ding H, Piao L, Joseph Huang S. Factors associated with successful transabdominal sonography-guided dilation and curettage for early cesarean scar pregnancy. Int J Gynaecol Obstet. 2015;131(3):281-4.

96. Zhai JF, Xu M, Zhang B, Gao JW, Chen N. Treatments of caesarean scar pregnancy and the corresponding results in ten years. Eur Rev Med Pharmacol Sci. 2015;19(14):2523-7.

97. Zhu X, Deng X, Wan Y, Xiao S, Huang J, Zhang L, et al. High-intensity focused ultrasound combined with suction curettage for the treatment of cesarean scar pregnancy. Medicine (Baltimore). 2015;94(18):e854.

98. Cao S, Zhu L, Jin L, Gao J, Chen C. Uterine artery embolization in cesarean scar pregnancy: safe and effective intervention. Chin Med J (Engl). 2014;127(12):2322-6.

99. Gao L, Huang Z, Gao J, Mai H, Zhang Y, Wang X. Uterine artery embolization followed by dilation and curettage within 24 hours compared with systemic methotrexate for cesarean scar pregnancy. Int J Gynaecol Obstet. 2014;127(2):147-51.

100. He Y, Wu X, Zhu Q, Wu X, Feng L, Wu X, et al. Combined laparoscopy and hysteroscopy vs. uterine curettage in the uterine artery embolization-based management of cesarean scar pregnancy: a retrospective cohort study. BMC Womens Health. 2014;14:116.

101. Li YR, Xiao SS, Wan YJ, Xue M. Analysis of the efficacy of three treatment options for cesarean scar pregnancy management. J Obstet Gynaecol Res. 2014;40(11):2146-51.

102. Shao H, Ma J, Su X, Xu L, Yang C, Su X, et al. Study of individualization therapy for 61 patients with cesarean scar pregnancy. Clin Exp Obstet Gynecol. 2014;41(5):551-5.

103. Wang G, Liu X, Bi F, Yin L, Sa R, Wang D, et al. Evaluation of the efficacy of laparoscopic resection for the management of exogenous cesarean scar pregnancy. Fertil Steril. 2014;101(5):1501-7.

104. Di Spiezio Sardo A, Zizolfi B, Saccone G, Ferrara C, Sglavo G, De Angelis MC, et al. Hysteroscopic resection vs ultrasound-guided dilation and evacuation for treatment of cesarean scar ectopic pregnancy: a randomized clinical trial. Am J Obstet Gynecol. 2023;229(4):437.e1-.e7.

105. Yu L, Yang B, Xu Q, Teng Y, Xue Z. A study on the timing of uterine artery embolization followed by pregnancy excision for cesarean scar pregnancy: a prospective study in China. BMC Pregnancy Childbirth. 2021;21(1):697.

106. Li Y, Gong L, Wu X, Gao H, Zheng H, Lan W. Randomized controlled trial of hysteroscopy or ultrasonography versus no guidance during D&C after uterine artery chemoembolization for cesarean scar pregnancy. Int J Gynaecol Obstet. 2016;135(2):158-62.

107. Peng P, Gui T, Liu X, Chen W, Liu Z. Comparative efficacy and safety of local and systemic methotrexate injection in cesarean scar pregnancy. Ther Clin Risk Manag. 2015;11:137-42.

108. Qian ZD, Huang LL, Zhu XM. Curettage or operative hysteroscopy in the treatment of cesarean scar pregnancy. Arch Gynecol Obstet. 2015;292(5):1055-61.
